# A Rare Case of Chronic Pancreatitis With Giant Branching Pancreatic Calculi and Large Pancreatic Pseudocysts: Diagnostic and Surgical Challenges

**DOI:** 10.7759/cureus.95379

**Published:** 2025-10-25

**Authors:** Duy Khanh Dao, Valery Remirovich Goltsov, Dmitry Alexandrovich Surov

**Affiliations:** 1 Department of Naval Surgery, S. M. Kirov Military Medical Academy, Saint Petersburg, RUS

**Keywords:** chronic pancreatitis, coral-like stones, pancreatic duct stones, pancreatic pseudocyst, surgical treatment

## Abstract

Pancreatic pseudocysts and ductal calculi are common complications of chronic pancreatitis (CP), with the management of pancreatic duct stones posing particular challenges due to complex ductal anatomy and a high risk of recurrence and procedural complications. We present the case of a female patient with long-standing CP who was admitted with severe, persistent abdominal pain refractory to nonsteroidal anti-inflammatory drug (NSAID) therapy and a 10-kg weight loss over the two months preceding hospitalization. Diagnostic imaging revealed pseudocysts in the head and body of the pancreas and giant “coral-like” calculi obstructing the main pancreatic duct. The patient underwent surgical treatment consisting of a Berne (modified Beger) procedure with pancreaticojejunostomy. The postoperative course was uneventful, with no significant complications. Treatment efficacy was evaluated using laboratory parameters, imaging findings, and clinical outcomes. At discharge, the patient reported only mild residual pain and was released in satisfactory condition. This case is notable for the presence of giant pancreatic duct calculi measuring 30 × 35 mm and 32 × 12 mm - dimensions that are exceptionally rare in the literature.

## Introduction

Chronic pancreatitis (CP) is a progressive fibro-inflammatory disorder of the pancreas that causes irreversible structural and functional damage, with persistent abdominal pain being the predominant symptom leading to significant quality of life (QoL) impairment [1]. One of the characteristic morphological features of CP is the formation of calculi within the ductal system [2]. Large pancreatic duct stones may cause ductal obstruction, resulting in clinical manifestations of exocrine and/or endocrine pancreatic insufficiency, as well as persistent postprandial abdominal pain [3].

Patients with CP often present with dyspeptic symptoms and frequently adopt restrictive eating behaviors, often described as adherence to a “strict diet,” which may lead to malnutrition, anorexia, cachexia, and astheno-neurotic syndrome. Pancreatic calculi are considered a pathognomonic sign of CP, occurring in 50-90% of patients, particularly in those with long-standing disease [4-6]. Over the past two decades, at the hospitals of the Kirov Military Medical Academy and its affiliated centers, over 3,000 patients with CP have been registered. Among them, about 420 patients were diagnosed with chronic calcific pancreatitis, and large obstructing calculi (up to 15 mm in diameter) within the main pancreatic duct (MPD) were identified in nearly half of them. Surgical interventions, including the Partington-Rochelle, Frey, Beger, and other procedures, were performed in 168 patients to relieve ductal obstruction, remove large calculi, and decompress the pancreatic ductal system.

Here, we report an exceptionally rare case of CP complicated by giant staghorn-like pancreatic calculi and a large pancreatic pseudocyst. This case underscores the diagnostic complexity and surgical challenges associated with managing giant pancreatic duct stones in advanced CP.

## Case presentation

A 52-year-old female patient was admitted to the Department of Naval Surgery (General Surgery), Kirov Military Medical Academy, St. Petersburg, Russia, with complaints of recurrent abdominal pain, predominantly in the epigastric region, radiating to the back, and associated with generalized weakness. She had been diagnosed with chronic calcific pancreatitis in 2017 following an abdominal ultrasound, which revealed MPD dilatation up to 8 mm, although she was asymptomatic at that time. Since 2021, she has had type 2 diabetes mellitus requiring insulin therapy. From August 2024, the patient began experiencing recurrent episodes of severe abdominal pain and was repeatedly hospitalized for exacerbations of CP. In November 2024, she underwent endoscopic retrograde cholangiopancreatography (ERCP) with endoscopic sphincterotomy (EST) and biliary stenting for biliary obstruction. However, attempts at stone extraction and intraductal mechanical lithotripsy were unsuccessful due to the large size and deep impaction of the calculi within the pancreatic parenchyma. During the following months, she continued to experience persistent epigastric pain, anorexia, early satiety, and significant weight loss (>10 kg within two months). She had been using nonsteroidal anti-inflammatory drugs (NSAIDs) long-term for pain control. Ultrasonography performed in February 2025 revealed progression of a pancreatic cyst measuring up to 8 cm in diameter.

On examination, the patient was hemodynamically stable, with vital signs within normal limits. Her body mass index (BMI) was 19.1 kg/m², with asthenic body habitus and signs of malnutrition. Physical examination revealed approximately an 8 × 8 cm epigastric mass of firm-elastic consistency. Laboratory tests showed anemia (Hb 97.4 g/L; reference: 120-160 g/L), hypoproteinemia (total protein 58.4 g/L; reference: 65-85 g/L), elevated transaminases (AST 250.1 U/L, ALT 241.1 U/L; reference for both: <40 U/L), and markedly elevated alkaline phosphatase (1158 U/L; reference: 40-130 U/L). HbA1c was 7.1% (reference: <5.7%).

**Figure 1:**
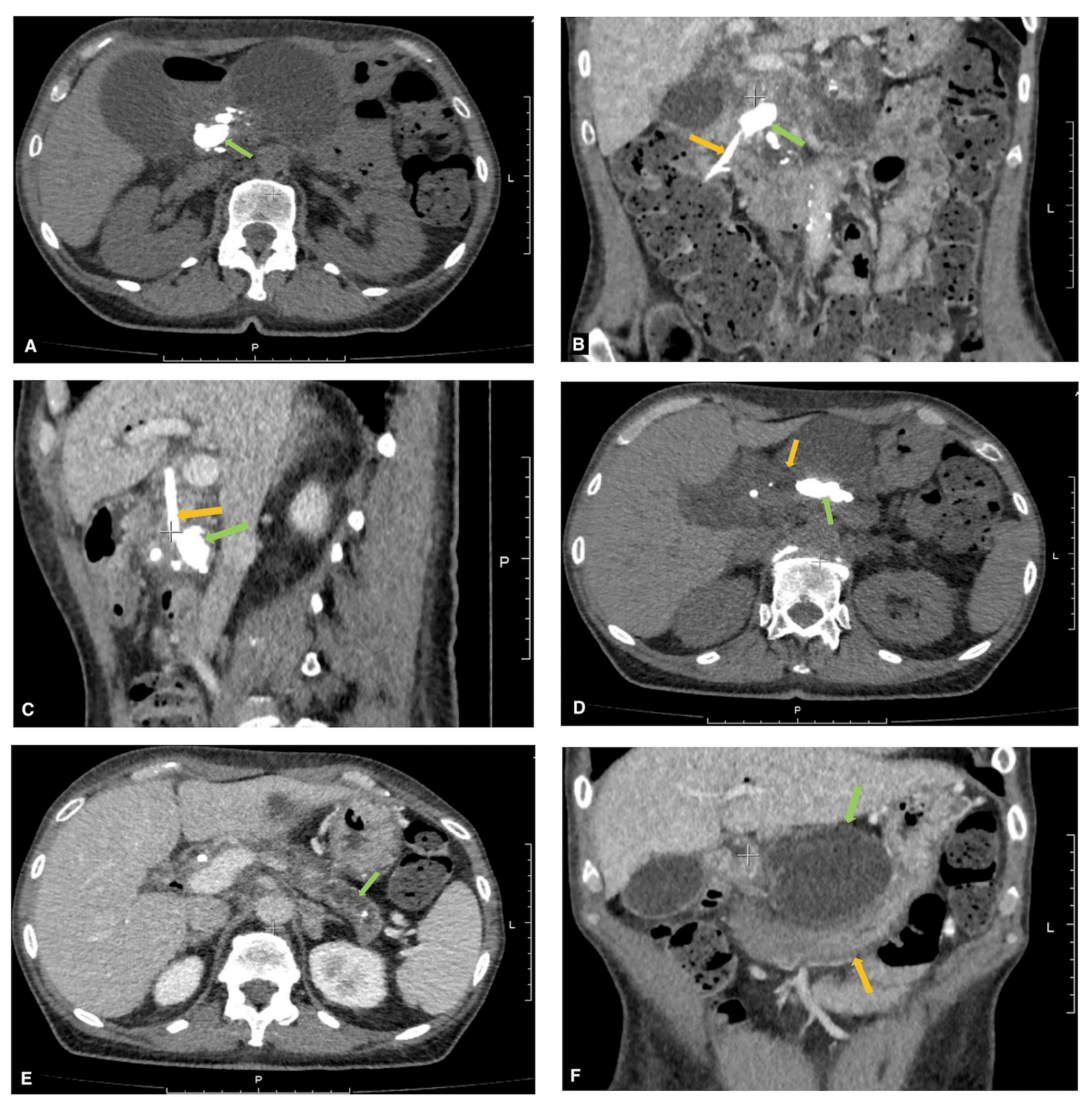
IV contrast-enhanced computed tomography (CT) findings of the abdomen A, B, C) A giant “coral-like” calculus (green arrow) 30 x 35 mm in the head of the pancreas with close contact to the endobiliary stent (orange arrow). D) A giant “coral-like” calculus (green arrow) 32 x 12 mm in the body of the pancreas. The MPD (orange arrow) between the head and body of pancreas is dilated up to 12 mm. E) The MPD (green arrow) distal to the body calculus appears tortuous and dilated (10-12 mm) in the body and tail of the pancreas. F) A pancreatic cyst (green arrow) in the body of the pancreas causing gastric compression (orange arrow).

Abdominal ultrasonography revealed a cystic lesion measuring 7.6 × 7.8 cm located in the body and isthmus of the pancreas. Triphasic contrast-enhanced computed tomography (CT) (Figure 1) demonstrated a giant coral-like calculus measuring 30 × 35 mm in the pancreatic head, which destroyed the MPD and formed a 35 × 40 mm cystic cavity. A second giant coral-like calculus measuring 32 × 12 mm was identified in the pancreatic body. The MPD distal to the obstruction was dilated to 10-12 mm. 

**Figure 2:**
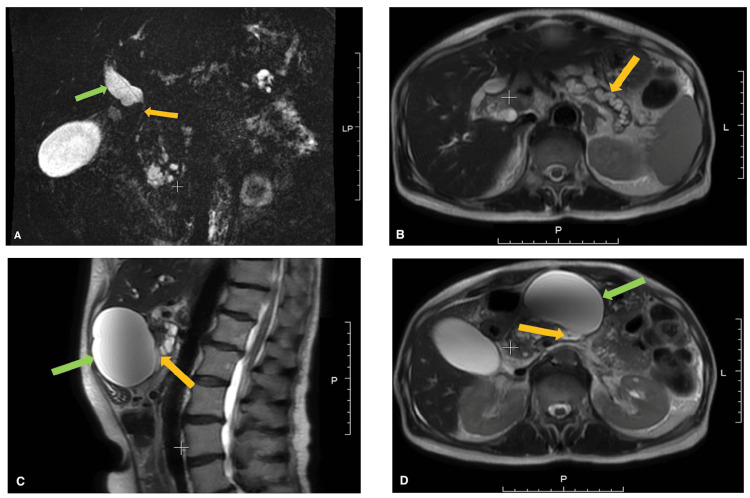
Magnetic resonance imaging findings A) MRCP (Magnetic Resonance Cholangiopancreatography): tubular bile duct stricture 35 mm in length (yellow arrow) with biliary hypertension, diameter of the common bile duct up to 15 mm (green arrow); B) MRI, axial view. Arrow marks a dilated main pancreatic duct (MPD) with a diameter of 12 mm; C) MRI, sagittal view. Green arrow shows pancreatic cyst in the body of pancreas with its communication (yellow arrow) with the MPD; C) MRI, axial view. Green arrow shows pancreatic cyst in the body of pancreas with its communication (yellow arrow) with the MPD.

Magnetic resonance imaging and cholangiopancreatography (MRI/MRCP) (Figure 2) revealed marked biliary and pancreatic ductal obstruction, with dilatation of the common bile duct (CBD) up to 15 mm and the MPD up to 12 mm. A 76 × 78 mm cyst was visualized in the pancreatic body, compressing the posterior wall of the stomach. A persistent distal CBD stricture was noted despite prior stenting performed four months earlier. Esophagogastroduodenoscopy (EGD) confirmed bile flow through the endobiliary stent, indicating maintained stent patency.

The preoperative diagnosis was chronic calcific pancreatitis complicated by ductal hypertension, characterized by giant coral-like calculi (30 × 35 mm and 32 × 12 mm), a pancreatic head cyst (35 × 40 mm), a pancreatic body pseudocyst (80 × 80 mm), dilatation of the MPD (8-12 mm), and biliary obstruction associated with CBD dilatation measuring 15 mm. Comorbidities included type 2 diabetes mellitus, anemia, hypoproteinemia, NSAID-induced hepatopathy, and astheno-neurotic syndrome.

**Figure 3:**
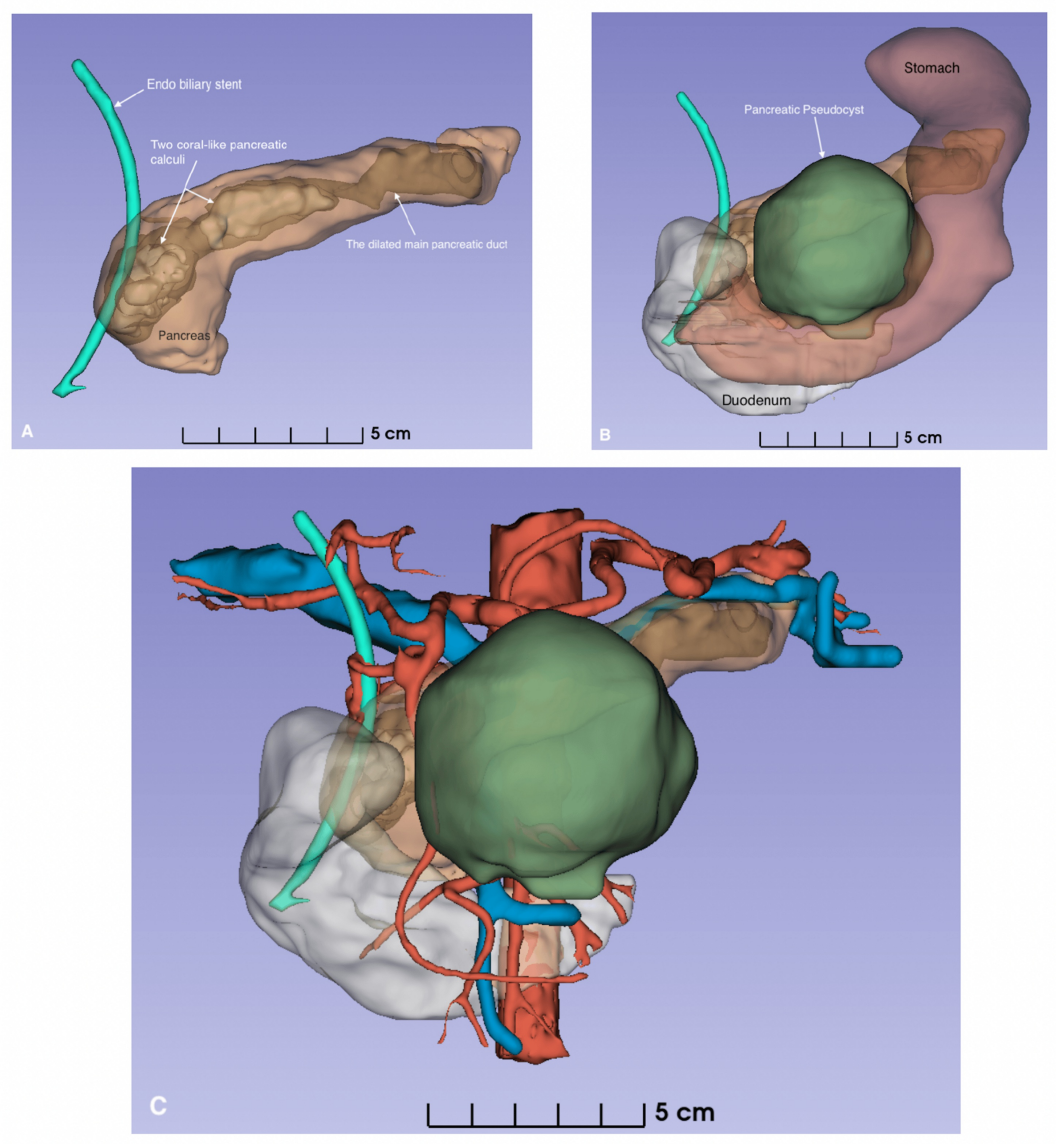
3D model of the pancreas reconstructed from CT (by using 3D-Slicer software) A) anterior view of the pancreas; B) anatomical syntopy of the stomach and pancreatoduodenal complex with the pancreatic pseudocyst; C) pancreatoduodenal complex with its vessels.

Owing to hepatotoxicity resulting from prolonged NSAID use, surgery was postponed to allow stabilization of liver function and nutritional optimization. Three-dimensional (3D) reconstruction using DICOM-based imaging data processed in “3D Slicer software” was utilized for surgical planning, providing precise anatomical visualization and enabling calculation of the planned pancreatic head resection volume (Figure 3).

**Figure 4:**
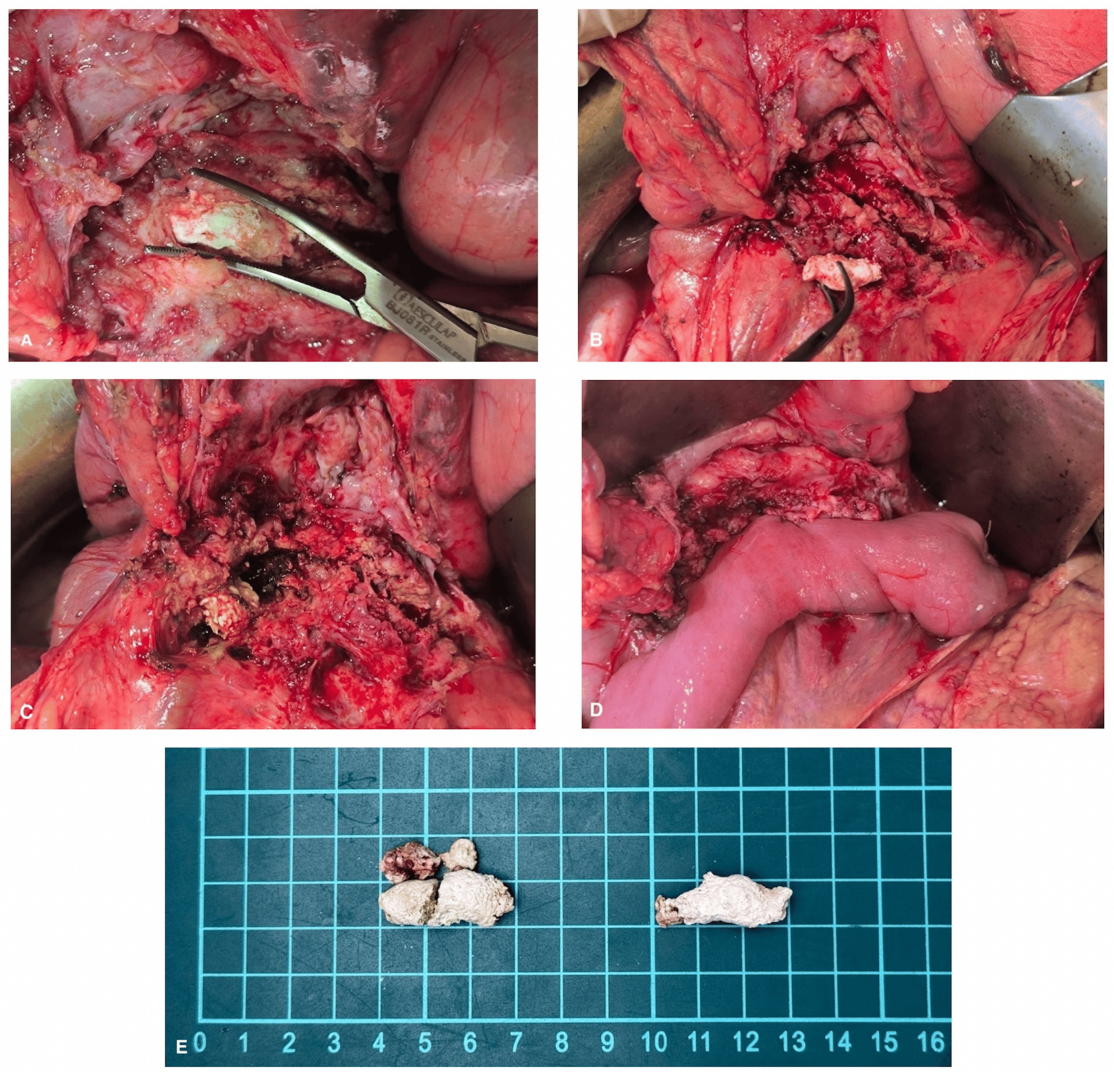
The Berne (modified Beger) procedure intraoperative findings A) giant coral-like calculus in the body of pancreas; B) dilated MPD; C) residual coral-like calculus fragments in the head of pancreas with biliary fluid leakage after subtotal resection; D) pancreaticojejunostomy; E) comparative fragments arrangement: left - fragments of giant coral-like calculus from the head of pancreas, right - from the body of the pancreas.

After 10 days of hospitalization, the patient underwent surgery. A midline laparotomy was performed under general anesthesia. Dense adhesions within the omental bursa were carefully lysed. A large cyst measuring 80 × 80 mm was identified in the pancreatic body, tightly adherent to the posterior gastric wall, and containing approximately 200 mL of hemorrhagic fluid. A Kocher maneuver was subsequently performed. The MPD was opened along the body and tail. The first giant coral-like calculi located in the pancreatic body were fragmented and removed with considerable difficulty. Proximally, a giant coral-like calculus in the pancreatic head was meticulously fragmented and extracted. Bile leakage from the posterior aspect of the pancreatic head confirmed communication with the biliary tree. To relieve biliary obstruction, a Beger-type pancreatic head resection with Roux-en-Y pancreaticojejunostomy (Bern modification) was performed, with the biliary stent left in place prophylactically (Figure 4).

The postoperative course was uneventful. No signs of postoperative pancreatitis, pancreaticojejunostomy (PJA) leakage, or postoperative pancreatic fistula (POPF) were observed. Oral feeding was resumed on postoperative day 3, and the patient was discharged in satisfactory condition on postoperative day 21. Final laboratory investigations prior to discharge demonstrated a steady improvement in liver function tests and biochemical parameters (Table 1).

**Table 1:** Summary of the laboratory values Hb: hemoglobin; AST: aspartate aminotransferase; ALT: alanine aminotransferase; ALP: alkaline phosphatase

Laboratory test and normal range	Preoperative value	Follow-up	Discharge value	After one month
Hb (g/L): 120-160	97 ↓	113 ↓	112 ↓	120
Serum proteins (g/L): 66-83	58 ↓	47 ↓	-	66
AST (U/L): 0-35	333 ↑	11	7	40
ALT (U/L): 0-35	358 ↑	15	9	36
ALP (U/L): 30-120	1158 ↑	238 ↑	140	120

Following hospital discharge, no evidence of pancreatic exocrine insufficiency was observed. The intensity of abdominal pain was markedly reduced compared with the preoperative level (3 vs. 9 on the visual analogue scale (VAS)). Histopathological examination of the resected pancreatic tissue confirmed chronic calculous pancreatitis. The tissue demonstrated dense sclerotic stroma with areas of fibrosis and hyalinization, mild lymphocytic infiltration, and dilated pancreatic ducts containing intraluminal calcifications. Foci of acinar atrophy were observed, consistent with chronic inflammatory remodeling. No dysplasia or malignant transformation was identified.

## Discussion

The term “coral-like calculus” is traditionally used in urology. However, given the striking resemblance of these pancreatic stones to coral-like branching renal calculi, the term is appropriate in this context. Giant coral-like pancreatic stones represent a rare and poorly documented manifestation of CP. To our knowledge, pancreatic duct stones with branching morphology and dimensions approaching 30 mm have been only sporadically reported. A review of the literature identified only isolated case reports describing CP complicated by giant coral-like pancreatic calculi [4,7,8]. Among them, Q. Liu et al. (2018) reported a 25 × 10 mm pancreatic duct stone - the largest previously documented - which was successfully removed using ERCP [4]. In that case, endoscopic extraction was possible because the stone was located in the proximal part of MPD, with a portion protruding through a major duodenal papilla into the duodenal lumen and visible during EGD. By contrast, in our case, the calculi were deeply embedded within the MPD, making endoscopic extraction technically impossible; therefore, surgical intervention was selected as the definitive treatment.

The principal etiological factors in chronic calcific pancreatitis include chronic alcohol consumption, gallstone disease, and genetic susceptibility [7]. According to the necrosis-fibrosis sequence theory, inflammatory necrosis is followed by periductal scarring, ductal stricture, impaired outflow of pancreatic secretions, and subsequent intraductal stone formation [9].

The choice of treatment depends on the stone size, number, location, and the presence of associated complications. Small calculi (≤5 mm), particularly those located in the proximal MPD, can often be successfully removed endoscopically. By contrast, large (>5 mm) or coral-like calculi typically require surgical intervention [4,10]. The primary goals of surgical methods are the removal of calculi obstructing the duct, decompression of the dilated pancreatic ducts, and preservation of the pancreatic parenchyma and nearby organs [11].

The present case is unique in demonstrating giant pancreatic duct calculi measuring 30 × 35 mm and 32 × 12 mm. Their formation likely reflects long-standing CP, chronic alcohol abuse, and delayed access to specialized surgical management. Minimally invasive procedures such as ERCP, endoscopic, or percutaneous drainage of pancreatic pseudocysts are generally inappropriate in such cases, as they carry a high risk of recurrence, fail to address the primary cause of pain - the ductal obstruction and pancreatic stones - and may exacerbate pancreatic fibrosis [11]. According to a recent systematic review and meta-analysis by Na et al. (2024), surgical intervention provides superior pain relief compared with endoscopic therapy in patients with chronic calcific pancreatitis [12].

## Conclusions

This report describes a rare case of CP complicated by giant coral-like pancreatic duct calculi (30 × 35 mm and 32 × 12 mm) and a large pancreatic pseudocyst. The patient underwent successful surgical management using the Berne (modified Beger) procedure, with an uneventful postoperative course and marked pain reduction. Giant coral-like pancreatic calculi represent a rare and challenging manifestation of CP that typically necessitates radical resectional surgery. Timely radical surgical intervention, such as the Frey-Beger procedures, can significantly improve patient outcomes and QoL. Successful postoperative recovery and long-term disease control require a comprehensive multidisciplinary approach involving gastroenterologists, pancreatic surgeons, endocrinologists, nutritionists, and psychotherapists. Despite advances in pancreatic surgery, the management of CP complicated by giant coral-like calculi remains a substantial clinical and surgical challenge that requires further study.

## References

[1] Dankha R, Sparrelid E, Gilg S, Löhr JM, Ghorbani P (2025). Surgical management of chronic pancreatitis: a narrative review. United European Gastroenterol J.

[2] Meyer A, Koszka AJ, Abreu P, Ferreira R, Fantauzzi MC, Segatelli V, David AI (2020). Chronic pancreatitis with ductal stones in the pancreatic head treated by surgery: a case report. J Surg Case Rep.

[3] Ito K, Takuma K, Okano N (2025). Current status and future perspectives for endoscopic treatment of local complications in chronic pancreatitis. Dig Endosc.

[4] Liu Q, Wang Y, Zeng H, Hu B (2018). Successful endoscopic removal of a rare, large impacted pancreatic duct stone using grasping forceps: a case report with video. Medicine (Baltimore).

[5] Paramythiotis D, Karlafti E, Kollatou AS (2024). Pancreatolithiasis: does management depend on clinical manifestations?. Am J Case Rep.

[6] Bush N, Tandan M (2025). Pancreatic duct calculi: pathophysiology and management. Curr Opin Gastroenterol.

[7] Oleksandr K, Danilenko I, Smorodska O (2018). Morphological and crystal chemical characteristic of panсreatic lithiasis. Wiadomosci lekarskie.

[8] Krieger AG, Karmazanovsky GG, Smirnov AV (2017). Ray diagnostics and surgical tactics for chronic pancreatitis [Article in Russian]. Khirurgiia (Mosk).

[9] Panek-Jeziorna M, Wierzbicki J, Annabhani A, Paradowski L, Mulak A (2017). Pancreatic duct stones: a report on 16 cases. Adv Clin Exp Med.

[10] Brebu D, Prodan-Bărbulescu C, Braicu V (2024). Surgical treatment of lithiasis of the main pancreatic duct: a challenging case and a literature review. Diseases.

[11] Kaushik N, Dasari V, Jain D (2023). Management of pancreatic calculi in chronic pancreatitis: a review article. Cureus.

[12] Na C, He T, Khalaf K (2024). Efficacy of endoscopic interventions versus surgery for pain management in patients with chronic calcific pancreatitis: a systematic review and meta-analysis. Surg Endosc.

